# The Evolution of Treatment Policies and Outcomes for Patients Aged 60 and Older with Acute Myeloid Leukemia: A Population-Based Analysis over Two Decades

**DOI:** 10.3390/cancers16233907

**Published:** 2024-11-21

**Authors:** Benno Diekmann, Nic Veeger, Johanne Rozema, Robby Kibbelaar, Bas Franken, Yasemin Güler, Bram Adema, Eric van Roon, Mels Hoogendoorn

**Affiliations:** 1 Unit of Pharmacotherapy, Epidemiology and Economics, Department of Pharmacy, Faculty of Science and Engineering, University of Groningen, 9713 AV Groningen, The Netherlands; 2 Department of Clinical Pharmacy and Pharmacology, Leeuwarden Medical Centre, 8934 AD Leeuwarden, The Netherlands; 3 Department of Internal Medicine, Leeuwarden Medical Centre, 8934 AD Leeuwarden, The Netherlands; 4 Academy, Leeuwarden Medical Centre, 8934 AD Leeuwarden, The Netherlands; 5 Department of Clinical Pharmacy and Pharmacology, University Medical Centre Groningen, 9700 AD Groningen, The Netherlands; 6 Department of Pathology, Leeuwarden Medical Centre, 8934 AD Leeuwarden, The Netherlands

**Keywords:** acute myeloid leukemia, AML, myelodysplasia, MDS, cytarabine, azacitidine, decitabine, venetoclax, stem cell transplantation, European Leukemia Net, population based

## Abstract

Background: Acute myeloid leukemia (AML) is a malignancy of the bone marrow with a median age at diagnosis of 70 years. AML is difficult to treat, especially in older patients, among whom outcomes have historically been poor. Over the last two decades, a greater understanding of the molecular mechanisms of the pathology has led to the development of new drugs and multiple updates to treatment guidelines. Methods: A population-based retrospective cohort study was conducted for all patients aged 60 and older who were newly diagnosed with AML (n = 370) as defined by the European Leukemia Net 2022 criteria in Friesland, a Dutch province, between 2005 and 2023. Results: In this cohort of patients with a median age of 73 years, complete bone marrow analysis to classify the AML according to ELN increased in time from 49% (2005–2011) to 86% (2022–2023). The rate of patients receiving antileukemic therapy increased over time (2005–2011: 19%; 2012–2016: 64%; 2017–2021: 75%; 2022–2023: 74%), mainly driven by the introduction of hypomethylating agents. Over these time periods, the use of intensive chemotherapy (13%, 27%, 27%, and 5%) and rates of stem cell transplantation (3%, 9%, 27%, and 14%) underwent similar development as more patients were deemed eligible for these interventions from 2012 onwards, but usage declined again after the introduction of venetoclax in 2022. The median overall survival was 3.7, 7.3, 8.0, and 9.4 months over the four time periods, respectively. Conclusions: Our study demonstrates how outcomes of patients with newly diagnosed AML aged 60 and older improved over the last two decades.

## Simple Summary

Acute myeloid leukemia is a malignancy of the bone marrow that is predominantly diagnosed in older patients and has historically been difficult to treat. Over the last two decades, a greater understanding of molecular pathology has led to multiple changes in the treatment landscape, reflected in both international and Dutch treatment guidelines. In this retrospective population-based cohort study, we analyzed how changes impacted first-line therapy choices, remission rates, and the survival of patients aged 60 and older with acute myeloid leukemia.

## 1. Introduction

Acute myeloid leukemia (AML) is a malignancy of the bone marrow with a median age at diagnosis of 70 years. AML is considered difficult to treat, especially in older patients, among whom outcomes have historically been poor [1,2]. Our understanding of the pathology of AML has deepened considerably over the last two decades, leading to multiple changes in the treatment landscape and policy in the past and presumably the future. Thus far, three developments stand out. First, age at diagnosis used to be a key factor to consider. It has since been replaced by an aggregate of more detailed measures of a patient’s fitness. Second, AML has seen gradual changes to its classification systems, moving away from being morphology-based and towards mutation-based in the World Health Organization (WHO) classification systems of 2008 and 2016; the International Consensus Classification (ICC) systems of 2012, 2017, and 2022; and the European Leukemia Network (ELN) risk classification systems of 2010, 2017, and 2022 [3,4,5,6,7,8]. Third, hypomethylating agents (HMAs), such as azacitidine (AZA) and decitabine (DEC), have become important treatment options for the first-line induction therapy of AML, especially in the elderly [9,10,11,12]. Since 2022, adding the BCL-2 inhibitor venetoclax (VEN) to the HMA backbone has increasingly been regarded as the new standard of care in the Netherlands. Furthermore, a multitude of drugs with antileukemic activity are currently in phase I-III clinical trials [13,14,15,16,17,18]. Most of these upcoming agents are targeted therapies and, hence, are only effective when the targeted mutation is present, contributing to the rising emphasis on mutation profile and Next-Generation Sequencing (NGS) techniques [8,13,19,20,21].

When a novel agent has proven its added value in phase III randomized control trials (RCTs) against the standard of care, changes to treatment guidelines and reimbursement policies may become appropriate. The real-world setting in which these agents then have to prove their efficacy again may, however, deviate from the phase III trial setting [22]. Patients in daily clinical practice may present with more comorbidities and a higher degree of frailty than the patients included in the RCTs [23,24]. The translation of RCT results into real-world settings is further complicated by local preferences and policies, in-between hospital idiosyncrasies, and advancements in supportive care over time. As patent-holder-designed phase III RCTs often compare one regimen against another instead of a combination of approaches against a combination of approaches, real-world studies are looked upon to evaluate the post-clinical effectiveness of novel agents and the impact of guideline changes [22,25]. In this population-based study, we aim to provide real-world data on first-line therapy choices made for primary induction therapy in older patients with AML under the various treatment policies of the past two decades and to show which endpoints were reached while doing so.

## 2. Materials and Methods

### 2.1. Study Design

A retrospective, population-based cohort study was conducted utilizing the database of the HemoBase consortium and electronic health records (EHRs) from four hospitals located in Friesland, a Dutch province with about 650,000 inhabitants [22]. The study population included all patients aged 60 years or older who were newly diagnosed with AML as defined by the ELN2022 criteria with >11% bone marrow myeloblasts in Friesland between 1 January 2005 and 31 December 2023 [26,27,28].

### 2.2. Stratification of the Inclusion Period

The inclusion time was stratified into four periods based on three cutoff dates. Changes to the AML treatment policy were sometimes abrupt and sometimes gradual. Cutoff dates were chosen to represent the most relevant policy changes while maintaining sufficient group size. The first cutoff date, 1 January 2012, represents the onset of using HMA monotherapy as a first-line therapy outside of the trial setting in the Frisian hospitals [29]. The second cutoff date, 1 January 2017, was chosen to represent the changes in WHO2016, ICC2017, and ELN2017 classification systems taking effect in clinical care. Although changes to the treatment landscape compared to before 2017 are small, changes in diagnostic procedures and decision-making warrant a separate analysis period. The third cutoff date, 1 January 2022, was chosen because reimbursement of VEN was approved for first-line therapy of AML in the Netherlands in early 2022. HMA+VEN combination regimens quickly became the default choice for the induction of patients ineligible for intensive chemotherapy thereafter, replacing HMA monotherapy.

### 2.3. Handling of the MDS-EB2 Population

The bone marrow blast percentage cutoff for a diagnosis of AML was reduced from 20% to 11% in the ELN2022 guideline, thereby removing MDS-EB2 as a diagnosis from the classification systems. Patients diagnosed with MDS-EB2 prior to 2022 were reclassified as de novo AML for the purpose of this study. The ELN cytogenetic risk classification was retrospectively applied to these patients based on the available cytogenetics and molecular risk data.

### 2.4. Collection of Baseline, Treatment, and Outcome Data

Baseline data including age, sex, diagnosis, WHO/ECOG score, medical history, and blood and bone marrow analysis results were extracted from the HemoBase database and enriched where necessary using the electronic healthcare records (EHRs) of the four Frisian hospitals. Patients who had a history of myelodysplasia, chronic myeloid leukemia, chronic myelomonocytic leukemia, chronic myeloproliferative neoplasm, etc., prior to their AML diagnosis were classified as secondary AML (sAML). AML was classified as therapy-related (tAML) if patients had previously undergone treatment with radiation therapy, alkylating agents (such as platinum compounds, cyclophosphamide, busulfan, or bendamustine), or topoisomerase inhibitors (such as etoposide). For patients diagnosed before 2017, the ELN2017 risk classification was applied retrospectively to the available data on cytogenetics and molecular aberrations. The retrospective assignment of the ELN2022 classification on top of the ELN2017 classification was waived as these systems differ mainly in a number of genes that were added to the adverse category that were rarely, if at all, measured before 2017. From 2021 onwards, a commercial 98-gene panel for myeloid malignancies from SOPHiA Genetics, Rolle, Switzerland was used for NGS profiling of bone marrow samples [30]. The completeness of medical records and patient history enabled the retrospective assessment of the Hematological stem Cell Transplant Comorbidity Index (HCT-CI) whenever not outright registered by the hematologist [31]. The choice of first-line therapy was registered. All cytarabine-based and HMA-based regimens were considered antileukemic therapy. Patients receiving no antileukemic therapy were considered as receiving the best supportive care (BSC), including those who had received hydroxycarbamide or mercaptopurine. Outcomes, including the dates of first achievement of complete remission with incomplete blood count recovery (CRi), complete remission (CR), allogenic hematologic stem cell transplant (HSCT), and death, were registered.

### 2.5. Endpoints and Statistical Analysis

The primary endpoints were the choice of first-line therapy, overall survival (OS), rates of early death (60-day mortality), and one- and two-year survival rates per period and per first-line therapy. Secondary endpoints included rates of patients accomplishing CRi, CR, and undergoing HSCT and the time from diagnosis until these events.

Survival differences between treatment periods, as well as the type of first-line therapy, were estimated using a Cox proportional hazards model. Additionally, survival was visualized using the Kaplan–Meier method, from which median overall survival and 1- and 2-year survival rates were derived. Follow-up was completed up to 31 August 2024, after which censoring was applied. Patients lost to follow-up before that date were censored on the last date they were seen by a healthcare practitioner. The overall follow-up time was estimated using the method of Schemper and Smith [32]. Data were analyzed using the Statistical Package for the Social Sciences from IBM, New York, NY, USA, version 28. A two-sided *p*-value < 0.05 was used to indicate statistical significance.

## 3. Results

**Figure 1:**
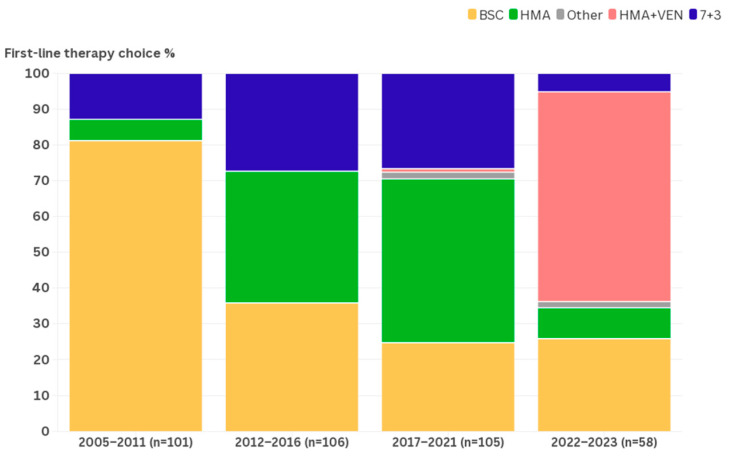
The proportion of first-line therapies chosen per period as given in the bar chart diagram [33].

**Figure 2:**
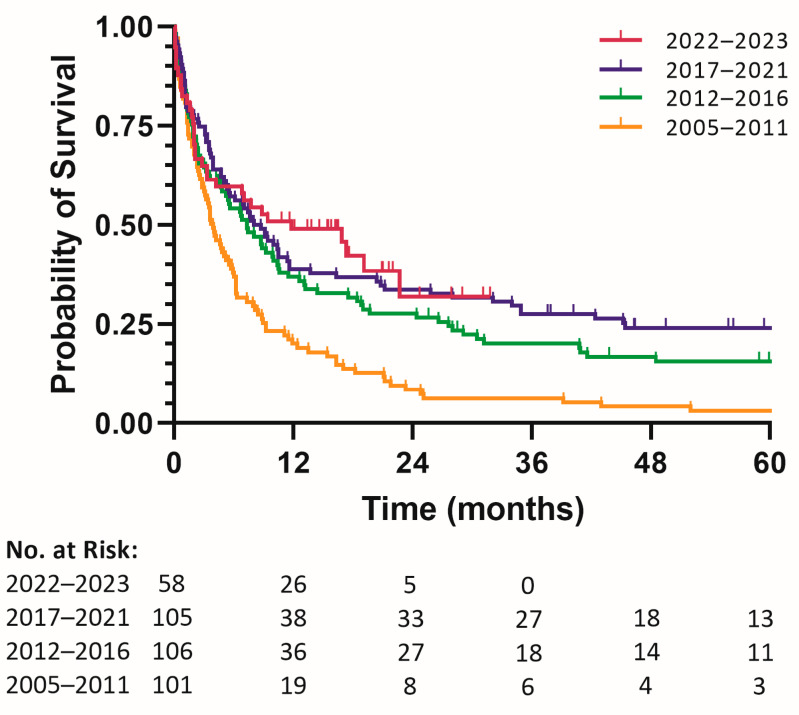
Kaplan–Meier survival analysis per period.

**Figure A1:**
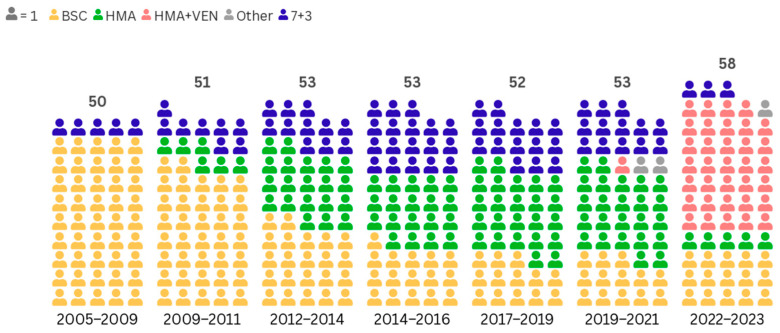
Patients and their first-line therapies in groups of ~50 consecutive patients [33].

A total of 370 patients with newly diagnosed AML were included, 36 of whom had originally been assigned the diagnosis MDS-EB2. The patients had a median age of 73 years, 19% had an impaired level of functioning of WHO 2–4, and 26% had considerable comorbidity (HCT-CI > 3). Of the patients whose bone marrow was analyzed, 56% percent had an AML with an ELN 2017 classification of adverse. Overall, 209 patients (56.5%) received antileukemic therapy and were followed for a median of 12.3 months. The median follow-up time in patients not receiving antileukemic therapy was 2.2 months. The baseline characteristics and first-line therapy choices are displayed in Table 1. Figure 1 shows the prevalence of the various first-line therapy choices over the four periods. A clear trend towards more patients undergoing antileukemic therapy (2005–2011: 19% vs. 2012 and after: 71%) can be observed. A detailed figure (Figure A1) is included in the Appendix A. Outcome data grouped per treatment period are presented in Table 2 and Figure 2.

**Figure 3:**
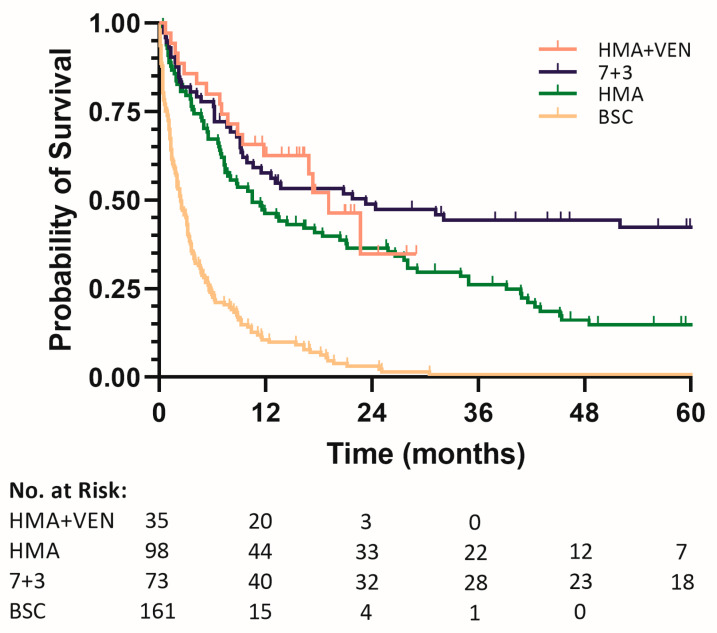
Kaplan–Meier survival analysis per first-line therapy.

The median overall survival was 3.7, 7.3, 8.0, and 9.4 months over the four periods. In the COX proportional hazards model, calendar time, stratified into four periods, was significantly associated with improved survival (*p* < 0.001). As more patients between 2012 and 2016 received antileukemic treatment (64% vs. 19%), rates of CR/CRi doubled compared to the 2005–2011 period (14% vs. 6%). Patients lived significantly longer (median OS = 7.3 vs. 3.7 months) and had a significantly lower risk of mortality (hazard ratio for death, 0.64; 95% confidence interval: 0.48–0.86, *p* = 0.003). The rates of patients undergoing antileukemic treatment (76%) and reaching CR/CRi (36%), as well as survival (median OS = 8.0 months), continued to rise in the 2017–2022 period (HR for death, 0.804, against 2012–2016; 95% CI: 0.592–1.091, *p* = 0.161; HR for death, 0.52, against 2005–2011; 95% CI: 0.38–0.70, *p* < 0.001). In the 2022–2023 period, no further rise in the proportion of patients undergoing treatment was observed (75%). Remission rates (CRi 66%) and survival (median OS = 9.4 months) were, however, improved upon (HR for death, 0.953, against 2017–2021; 95% CI: 0.634–1.433, *p* = 0.818; HR for death, 0.49, against 2005–2011; 95% CI: 0.33–0.73, *p* < 0.001). Table 3 and Figure 3 present the same endpoints, grouped by first-line therapy.

Patients treated with HMA (n = 98, 86 AZA, 12 DEC) as first-line therapy reached CR/CRi in 25% of cases. The survival of patients treated with HMA (median OS, 10.0 months) was improved compared to patients who were only provided BSC (HR for death, 0.32; 95% CI: 0.24–0.43, *p* < 0.001). No significant difference in OS or HR was found between patients treated with HMAs in the different periods. Patients who received HMA combined with VEN (n = 35, 8 AZA, 27 DEC) reached CR/CRi three times as often (77%) and survived longer than those without VEN (median OS, 17.3 months; HR against HMA, 0.65; 95% CI: 0.38–1.10, *p* = 0.11; HR against BSC, 0.21; 95% CI: 0.13–0.35, *p* < 0.001). The proportion of patients receiving HMA or HMA with VEN who proceeded to HSCT was 11% and 14%, respectively. In patients treated with standard 7+3 therapy, CR/CRi was observed most often (52%) and the quickest (median time to CR/CRi of 1.3 months). Consolidation with HSCT was observed in 42% of these patients. The 7+3 regimen resulted in superior survival compared to the other treatments (median OS = 20.7 months; HR against BSC, 0.18; 95% CI: 0.13–0.26, *p* < 0.001; HR against HMA, 0.56; 95% CI: 0.38–0.81; *p* = 0.002; HR against HMA+VEN, 0.86; 95% CI: 0.48–1.53, *p* = 0.608). No significant difference in OS or HR was found in patients treated with 7+3 in the different periods. In line with the observation of crossing survival graphs in Figure 3, no significance was reached in the comparisons of 7+3 against HMA+VEN and HMA+VEN against HMA.

Before 2012, complete bone marrow analysis was frequently omitted, likely due to the frailty of the patients and the lack of treatment modalities in that period. From 2017 onwards, bone marrow analysis was only rarely omitted, for example, in patients in a terminal health state at diagnosis. Overall, the completeness of baseline data improved throughout the time periods.

## 4. Discussion

### 4.1. Policy and Survival

In this population-based study representative of the real world, we demonstrate that patients aged 60 and older with newly diagnosed AML saw multiple shifts to first-line therapy in the last two decades. With increasing emphasis on mutational profiling, full bone marrow analysis developed from an exception in the 60+ patients in 2005–2011 to become the standard of care from 2017 onwards. By lowering the eligibility criteria for intensive chemotherapy, more patients were treated using this regimen, also leading to increasing rates of HSCT. From 2022 onwards, a shift from the intensive 7+3 induction chemotherapy to the HMA+VEN regimen could be observed. Ten days of decitabine (DEC10), having been shown to be equally as effective as 7+3 while being more tolerable, may have contributed to this trend [34]. In this population-based study representative of Dutch clinical practice, survival outcomes of patients aged 60 to 79 were improved by these policy changes. The outcomes of patients aged 80 and older, however, remain poor.

### 4.2. Distribution of Baseline Characteristics

The distribution of the baseline characteristics found is in line with other real-world studies and remained mostly stable throughout the included period [35,36,37,38]. Rates of patients with a WHO/ECOG score > 2 decreased from 2017 onwards, while the median age at diagnosis remained the same. This may hint at an improvement in the baseline fitness state of older patients in the Netherlands over time due to, e.g., healthy aging campaigns, which is in line with our subjective impression of the patients. Missing HCT-CI data (38% before 2017) likely occurred more frequently in patients in poorer health which may have masked a potential decrease in comorbidity burden.

An increasing proportion of AML classified as adverse based on the ELN2017 criteria was observed over the study period. This development is likely driven by changes in local policy regarding cytogenetic and molecular profiling as older patients with a higher prevalence of adverse risk profiles that were previously not assessed are now being assessed. A 6:3:1 ratio of the ELN2017 adverse to intermediate to favorable categories was observed in the overall study population, in line with other real-world studies of older patients with AML [11,35,37,39,40]. For patients from 2017 onwards, the ELN2022 classification could also be assessed, resulting in a 10:4:1 ratio, suggesting that changing classification systems can also contribute towards a shift to adverse classification [8].

### 4.3. Choice of First-Line Therapy

Since an age at diagnosis above 60, and later 65, was considered an eligibility criterion for intensive chemotherapy and HSCT, few patients underwent these trajectories before 2012. With consensus in the field shifting towards a combination of varying fitness criteria replacing age, more patients were recommended the 7+3 regimen. From 2012 onwards, many patients receiving the 7+3 regimen participated in clinical trials [34,41,42]. Parallel to the increased usage of the 7+3 regimen in patients aged 60 and older, HMAs were introduced as a less toxic alternative for first-line induction therapy. These developments coinciding resulted in a stark reduction in the number of patients who could only be offered BSC from 2012 onwards. Although the rate of patients aged 80 and older receiving antileukemic therapy increased considerably with the introduction of HMAs, median OS and rates of 1- and 2-year survival remained poor. Endpoints that were not measured, such as transfusion dependence and quality of life, may have nevertheless been improved in these patients. In the 2016–2021 period, the number of patients not receiving antileukemic therapy reduced to a quarter of diagnoses where it has since remained. By considering more patients as eligible for HMA monotherapy, a further but small increase in life expectancy could be realized. The overall CR/CRi rate of 25% and median OS of 10.0 months represent real-world outcomes for patients on HMA monotherapy [43,44]. Induction with the more intensive DEC10 regimen became an option for somewhat fitter patients who were still deemed ineligible for the 7+3 regimen, contributing to increased remission rates [34].

### 4.4. The Onset of Venetoclax

The VIALE-A trial results published in 2020 marked the first success in improving upon HMA monotherapy regimens [9]. In early 2022, the addition of VEN to an HMA-based induction therapy for AML was approved for reimbursement in the Netherlands. In Friesland, all patients were referred to the central hospital to undergo this novel treatment that was quickly adopted and consequently offered to most patients deemed ineligible for intensive chemotherapy. Concomitantly, in our region, DEC10 became the preferred HMA regimen to combine with VEN. In this way, a median OS of 17.3 months was achieved. The higher survival rate compared to the VIALE-A study may be explained by the inclusion of patients younger than 65 who were excluded from the RCT. Despite the high rate of CR/CRi after induction with HMA+VEN, the number of patients proceeding towards HSCT remains limited. The comorbidity burden in these patients hindering them from proceeding towards an HSCT partially explains the low number of HSCTs. The efficacy and tolerability of a regimen consisting of 5 days of HMA combined with 7 to 14 days of VEN, administered every 4–6 weeks as a maintenance therapy, likely also plays a role. Considering how many patients reach and maintain CR under HMA+VEN, it raises the question of whether consolidation with HSCT, the only curative option for AML, should be considered more often in these patients, even those aged 70 and older.

### 4.5. Strengths

With an annual incidence of 3.5 per 100,000 and a population of 640,000 to 650,000 followed over a 19-year inclusion period, a total of 425 cases of AML were expected to develop in our region [45]. Considering that patients younger than 60 were excluded, an inclusion amount of 370 patients with AML represents a high degree of completeness. Combined with the lack of exclusion criteria aside from age, our population-based study avoids most forms of selection bias while providing insight into the changes in diagnostic and treatment changes over two decades.

### 4.6. Limitations

The retrospective nature of this study in combination with the long inclusion period results in missing data. Missing baseline data are likely biased towards patients with poorer outcomes, who tend to have more missing data, especially in the 2005–2011 period. Missing outcome data due to patients being lost to follow-up were rare. Patients diagnosed in the 2022–2023 period who were still alive at the date of closing the database, however, set a ceiling for positive remarks on outcomes of this group. When making remarks on policy, it must be considered that treatment choices were made by humans in a shared decision-making process rather than purely algorithmically following current guidelines.

## 5. Conclusions

Our study illustrates that outcomes for patients aged 60 and older with newly diagnosed AML have improved over the last two decades. By switching from the age threshold for intensive chemotherapy to more detailed measures of a patient’s fitness, the rate of patients aged 60 and older undergoing this treatment doubled. The addition of HMA to the first-line therapeutic arsenal improved outcomes by enabling the treatment of patients who previously would have only received BSC. Later, the addition of VEN to HMA-based first-line therapy more than doubled the number of patients that were able to reach CR/CRi. The rate of patients aged 60 and older opting for intensive chemotherapy is decreasing, likely due to the effectiveness and tolerability of the competing HMA+VEN regimen. Rates of patients undergoing stem cell transplantation mirrored the increase and later decrease in the 7+3 regimen usage.

Future research may focus on identifying groups of patients that benefit more or less than average from these changes to guide further improvement and identify which patients in first CR after induction with HMA+VEN should proceed to allogeneic HSCT.

## Figures and Tables

**Table 1:** Population characteristics and chosen first-line therapies per treatment period.

Period	2005–2011	2012–2016	2017–2021	2022–2023	Total
**Demographics**					
n (%)	101 (27%)	106 (29%)	105 (28%)	58 (16%)	370 (100%)
Female, n (%)	45 (45%)	31 (30%)	33 (31%)	19 (33%)	128 (35%)
Age in years, median [Q1–Q3]	75 [69–80]	73 [66–78]	72 [68–78]	74 [70–77]	73 [68–78]
60–69 years, n (%)	30 (30%)	40 (38%)	36 (34%)	14 (24%)	120 (32%)
70–79 years, n (%)	44 (44%)	43 (41%)	49 (47%)	35 (60%)	171 (46%)
>80 years, n (%)	27 (27%)	23 (22%)	20 (49%)	9 (16%)	79 (21%)
**WHO/ECOG score**					
0–1, n (%)	62 (61%)	69 (65%)	88 (84%)	50 (86%)	269 (73%)
2–4, n (%)	22 (22%)	24 (23%)	15 (14%)	8 (14%)	69 (19%)
Unknown, n (%)	17 (17%)	13 (12%)	2 (2%)	0	32 (9%)
**HCT-CI**					
0, n (%)	21 (21%)	17 (16%)	19 (18%)	5 (9%)	62 (17%)
1, n (%)	12 (12%)	10 (10%)	10 (10%)	12 (21%)	44 (12%)
2–3, n (%)	11 (11%)	20 (19%)	39 (37%)	19 (33%)	89 (21%)
>3, n (%)	17 (17%)	21 (20%)	36 (34%)	22 (38%)	97 (26%)
Unknown, n (%)	40 (40%)	38 (36%)	1 (1%)	0	78 (21%)
**Diagnosis**					
De novo AML, n (%)	74 (73%)	77 (73%)	91 (87%)	41 (71%)	283 (76%)
sAML, n (%)	20 (20%)	17 (16%)	9 (9%)	12 (21%)	58 (16%)
tAML, n (%)	7 (7%)	12 (11%)	5 (5%)	5 (9%)	29 (8%)
**ELN2017 classification**					
Favorable, n (%)	4 (4%)	8 (8%)	9 (9%) ^C^	4 (7%) ^D^	25 (7%)
Intermediate, n (%)	16 (16%)	29 (27%)	28 (27%) ^C^	12 (21%) ^D^	85 (23%)
Adverse, n (%)	29 (29%)	34 (32%)	51 (49%) ^C^	34 (59%) ^D^	148 (40%)
Unknown, n (%)	52 (51%)	35 (33%)	17 (16%)	8 (14%)	112 (30%)
**First-line therapy**					
7+3 ^A^, n (%)	13 (13%)	29 (27%)	28 (27%)	3 (5%)	73 (20%)
BSC only, n (%)	82 (81%)	38 (36%)	26 (25%)	15 (26%)	161 (44%)
HMA mono, n (%)	6 (6%)	39 (37%)	48 (46%)	5 (9%)	98 (26%)
HMA+VEN, n (%)	0	0	1 (1%)	34 (59%)	35 (9%)
Other ^B^, n (%)	0	0	2 (2%)	1 (2%)	3 (1%)

^A^ Intensive chemotherapy, commonly consisting of 7 days of cytarabine with 3 days of any anthracycline per cycle. Patients treated in a ‘cytarabine with anthracycline versus cytarabine with anthracycline with another agent’ trial design were classified as 7+3; ^B^ two patients started on low-dose cytarabine and one on AZA with VEN with Magrolimab. ^C^ By ELN2022 classification, favorable: 6 (6%); intermediate: 24 (23%); and adverse: 58 (55%). ^D^ By ELN2022 classification, favorable: 3 (5%); intermediate: 10 (17%); and adverse: 37 (64%).

**Table 2:** Rates of CRi, CR, HSCT, and survival per period.

Period	2005–2011	2012–2016	2017–2021	2022–2023	Total
n (%)	101 (27%)	106 (29%)	105 (28%)	58 (16%)	370 (100%)
**Remission**					
Reached CRi, n (%)	N/A ^A^	N/A ^A^	21 (20%)	26 (45%)	47 (35%)
Time to CRi in months, median	N/A ^A^	N/A ^A^	1.9	1.2	1.6
Reached CR, n (%)	6 (6%)	15 (14%)	25 (24%)	10 (17%)	56 (15%)
Time to CR in months, median	1.5	2.6	1.3	1.0	1.6
Reached CR/CRi, n (%)	6 (6%)	15 (14%)	38 (36%)	31 (53%)	90 (24%)
Time to CR/CRi in months, median	1.5 ^A^	2.6 ^A^	1.5	1.2	2.0
**Transplantation**					
HSCT, n (%)	3 (3%)	9 (9%)	28 (27%)	8 (14%)	48 (13%)
Time to HSCT in months, median	4.2	3.8	3.8	3.8	4.0
**Survival**					
Overall survival in months, median	3.7	7.3	8.0	9.4	6.2
OS of 60–69 year olds, median	6.2	12.6	34.9	Not reached ^B^	13.5
OS of 70–79 year olds, median	4.6	5.4	7.7	11.8 ^B^	6.8
OS of 80+ year olds, median	2.8	5.6	2.5	1.9	2.5
1-year survival overall, %	19.6%	36.8%	38.1%	48.1% ^B^	34.3% ^B^
1-year survival 60–69, %	33.3%	51.9%	63.3%	71.4% ^B^	53.0% ^B^
1-year survival 70–79, %	19.3%	30.1%	34.0%	48.4% ^B^	32.3% ^B^
1-year survival 80+, %	4.0%	20.3%	0%	11.1%	8.5%
2-year survival, %	8.3%	27.6%	33.0%	31.4% ^B^	25.1% ^B^
2-year survival 60–69, %	20.0%	44.1%	57.5%	N/E ^B^	44.8% ^B^
2-year survival 70–79, %	4.8%	20.1%	27.6%	23.5% ^B^	20.3% ^B^
2-year survival 80+, %	0%	10.1%	0%	0%	2.8%
Early death, n (%)	33 (32.7%)	32 (30.2%)	25 (23.8%)	14 (24.1%)	104 (28.1%)
Follow-up time in months, median	3.6	6.1	7.4	10.1	5.6
Followed until death, n (%)	96 (95%)	88 (83%)	78 (74%)	34 (59%)	296 (80%)
**BSC**					
Received only BSC, n (%)	82 (81%)	38 (36%)	26 (25%)	15 (26%)	161 (44%)
Age in years, median [Q1–Q3]	76 [71–82]	76 [73–83]	80 [73–84]	78 [73–84]	78 [72–83]
Overall survival in months, median	2.9	1.6	2.1	0.7	2.2

N/A, not applicable; N/E, not estimable. ^A^ Achievement of CRi could not be assessed consequently for patients diagnosed before 2017; ^B^ 24 patients of the 2022–2023 period were still alive at the date of final analysis, 19 of which had been followed for less than two years.

**Table 3:** Rates of CRi, CR, HSCT, and survival per first-line therapy.

	BSC	HMA	HMA+VEN	7+3
**Period**				
Total, n (%)	161 (44%)	98 (23%)	35 (9%)	73 (20%)
2005–2011, n (%)	82 (81%)	6 (6%)	0	13 (13%)
2012–2016, n (%)	38 (36%)	39 (37%)	0	29 (27%)
2017–2022, n (%)	26 (25%)	48 (46%)	1 (1%)	28 (27%)
2022–2023, n (%)	15 (26%)	5 (9%)	34 (59%)	3 (5%)
**Remission**				
Reached CRi, n (%)	0	18 (18%) ^A^	23 (66%) ^A^	7 (10%) ^A^
Time to CRi in months, median	N/A	3.3 ^A^	1.2 ^A^	1.3 ^A^
Reached CR, n (%)	0	14 (14%)	8 (23%)	34 (47%)
Time to CR in months, median	N/A	3.4	1.7	1.3
Reached CR/CRi, n (%)	0	25 (25%) ^A^	27 (77%) ^A^	38 (52%) ^A^
Time to CR/CRi in months, median	N/A	3.4 ^A^	1.7 ^A^	1.3 ^A^
**Transplantation**				
HSCT, n (%)	1 (1%)	11 (11%)	5 (14%) ^B^	31 (42%)
Time to HSCT in months, median	3.8	4.8	4.1	3.7
**Survival**				
Overall survival in months, median	2.1	10.0	17.3	20.7
1-year survival, %	10.3%	46.2%	62.2% ^B^	56.9%
2-year OS, %	3.0%	36.5%	34.8% ^B^	48.2%
Early death, n (%)	76 (47.2%)	16 (16.3%)	3 (8.5%)	10 (13.6%)
Follow-up time in months, median	2.2	10.5	14.5	13.7

N/A, not applicable; ^A^ Achievement of complete remission with incomplete cell count recovery could not be assessed consequently for patients diagnosed before 2017, and 2017–2023 data are shown; ^B^ 18 patients were still alive at the date of final analysis, with most of them in remission and still receiving HMA+VEN as maintenance therapy.

## Data Availability

The data supporting the conclusions of this article will be made available by the authors upon request.
